# Subjective and Objective Appraisals of Self-Rated Health

**DOI:** 10.1093/geroni/igaf122.3847

**Published:** 2025-12-31

**Authors:** Abolade Oladimeji, Kristen Berg, Douglas Einstadter, Adam Perzynski

**Affiliations:** Samford University, Birmingham, Alabama, United States; Case Western Reserve University, Cleveland, Ohio, United States; Case Western Reserve University, Cleveland, Ohio, United States; Case Western Reserve University, Cleveland, Ohio, United States

## Abstract

**Introduction:**

Positive self-perceptions of aging are linked to better self-rated health, we examined the impact of clinical diagnoses (e.g., sleep disorders and comorbidities) and subjective factors (e.g., sleep appraisal, social and physical limitations) on self-rated health.

**Methods:**

Our cohort consisted of 1,523 older adults (65+) with at least two outpatient primary care visits and one Medicare Annual Wellness Visit. We used an ordered logistic regression model to predict self-rated health, which was rated on a five-point scale from “poor” to “excellent.”

**Results:**

The regression analysis showed that older adults with sleep difficulties (based on the PHQ-9 sleep item) had poorer self-rated health than those without any sleep difficulties. However, there was no significant difference between those with and without a diagnosed sleep disorder. This suggests that subjective sleep appraisal is associated with self-rated health, while sleep disorder diagnosis is not. We also found that higher social limitations were linked to poorer health, while increased physical activity was associated with better self-rated health. Higher comorbidity scores were also associated with poorer health outcomes. Also, African Americans reported lower self-rated health compared to Whites and others.

**Discussion:**

Our findings indicate that self-rated health is influenced by both subjective and objective factors, including perceived sleep difficulties, social and physical limitations, and physical health status (e.g., comorbidities). This study highlights the significance of physical functioning and social engagement in shaping perceived health among older adults

